# The epigenetic function of androgen receptor in prostate cancer progression

**DOI:** 10.3389/fcell.2023.1083486

**Published:** 2023-03-21

**Authors:** Takahiro Sawada, Yoshiaki Kanemoto, Tomohiro Kurokawa, Shigeaki Kato

**Affiliations:** ^1^ Graduate School of Life Science and Engineering, Iryo Sosei University, Fukushima, Japan; ^2^ Research Institute of Innovative Medicine, Tokiwa Foundation, Fukushima, Japan; ^3^ School of Medicine, Fukushima Medical University, Fukushima, Japan

**Keywords:** epigenetic, prostate cancer, androgens, chromatin reorganization, genome

## Abstract

Androgen and androgen deprivation (castration) therapies, including androgen receptor antagonists, are clinically used to treat patients with prostate cancer. However, most hormone-dependent prostate cancer patients progress into a malignant state with loss of hormone-dependency, known as castration (drug)-resistant prostate cancer (CRPC), after prolong androgen-based treatments. Even in the CRPC state with irreversible malignancy, androgen receptor (AR) expression is detectable. An epigenetic transition to CRPC induced by the action of AR-mediated androgen could be speculated in the patients with prostate cancer. Androgen receptors belongs to the nuclear receptor superfamily with 48 members in humans, and acts as a ligand-dependent transcriptional factor, leading to local chromatin reorganization for ligand-dependent gene regulation. In this review, we discussed the transcriptional/epigenetic regulatory functions of AR, with emphasis on the clinical applications of AR ligands, AR protein co-regulators, and AR RNA coregulator (enhancer RNA), especially in chromatin reorganization, in patients with prostate cancer.

## 1 Introduction

Prostate cancer is a disease with several symptoms suffering over a million of men worldwide with death of over 0.3 million patients. However, the disease is curable if diagnosed early, with improved overall long-term survival in patients. The early developmental stage of prostate cancer is dependent on androgen. Therefore, hormone deprivation therapy is effective to attenuate cancer development, while adverse effects including hot flashes, anorexia and osteopenia are often seen. As standard treatments, pharmacological and surgical methods are used. Although hormone deprivation therapy is effective at the early stages, later most prostate cancer cases are transited into a hormone-independent prostate cancer state known as castration (drug)-resistant prostate cancer (CRPC) (Scher et al., 2012; Attard et al., 2016; Li et al., 2016c). Chemotherapy is clinically successful against CRPC; however, some CRPC patients develop metastatic CRPC (mCRPC), making effective treatment difficult to achieve. Thus, inhibiting the transition from a hormone-dependent to hormone-independent state is necessary to improve the quality of life (QOL) of patients. However, studies on strategies to inhibit the transition process are still preliminary, and the molecular mechanisms are yet to be elucidated. Recently, clinical studies have identified the importance of androgen signaling and the expression of androgen receptor (AR) in the transition from a hormone-dependent to hormone-independent state (Henshall et al., 2001; Sharifi, 2013; Antonarakis et al., 2014). In this review, we discussed the epigenetic function of AR in androgen signaling and its role in the transition process from hormone-dependent to hormone-independent prostate cancer.

### 1.1 The transition from hormone-dependent to castration (drug)-resistant prostate cancer (CRPC)

Androgen deprivation drugs, such as androgen antagonists and enzyme inhibitors of androgen biosynthesis, are effective against prostate cancer in the early stages (Scher et al., 2012; Attard et al., 2016; Li et al., 2016c). For example, the AR antagonists bicalutamide and flutamide were developed to antagonize endogenous androgen-induced activation of the gene regulatory function of AR, and have been clinically applied. Despite promising results, the efficacy of bicalutamide and flutamide is generally poor, and prolong used can cause drug resistance (Rice et al., 2019). To overcome this issue, a third-generation nonsteroidal antiandrogen (NSAA), darolutamide, has been developed (Crawford et al., 2019). Recently, other classes of androgen antagonists (enzalutamide) have been successfully developed, with better clinical outcomes in patients with CRPC than the AR antagonists (Scher et al., 2012). At the molecular level, enzalutamide anchors AR in cytosol even after AR binding, thereby blocking the action of endogenous androgens in prostate tumors (Nakazawa et al., 2014). Overall, enzalutamide treatment has been clinically more effective than the traditional bicalutamide and flutamide treatments in patients with CRPC and mCRPC (Li et al., 2016c). Unlike the AR antagonists, enzalutamide is an enzymatic inhibitor for p450 17A1 (CYP17) capable of irreversibly blocking steroid conversion in the biosynthesis pathway of active androgens in prostatic tumor cells (Li et al., 2016c). Although both enzalutamide and abiraterone have shown high efficacy against prostate cancer in clinical trials, they are unable to entirely eradicate prostate cancer (Romanel et al., 2015; Wang et al., 2021). Moreover, a radiopharmaceutical drug called pluvicto (lutetium Lu 177, vipivotide tetraxetan) has recently been evaluated with clinical success (Neels et al., 2021), indicating that new generation drugs with similar or distinct modes of action with the androgen deprivation drugs may be effective against prostate cancer.

### 1.2 Epigenetic transition during prostate cancer development

Despite the clinical application of new-generation drugs, some patients with hormone (androgens) sensitive prostate cancer (HSPC) become insensitive to androgen deprivation drugs after prolonged treatment. The transition from HSPC to CRPC is irreversible and is accompanied by alterations in gene expression profile, which may affect the chromatin landscape or follow the altered chromatin landscape (Malik et al., 2015; Cyrta et al., 2020). Over the years, the molecular mechanisms underlying cancer development and progression have been extensively studied. For example, several aberrant events in cellular signaling and DNA-templated biological processes, including transcription, DNA replication, and repair, have been identified in cancers, including prostate cancer (Gavande et al., 2016; Elsesy et al., 2020; Zhang et al., 2020). The DNA repair process includes specific histone modifications and chromatin remodeling at damaged chromatin regions, and DNA repair-induced epigenetic modifications have been observed in patients receiving radiotherapy (Clouaire et al., 2018). Moreover, a dysfunction in the regulation of DNA repair may occur during cancer development (Torgovnick and Schumacher, 2015).

Consistent with the role of DNA methylation in the inactivation of local chromatin state, DNA hypermethylation was observed in the promoters of the genes *SOSTDC1* and *FLT4*, and was associated with prostate cancer development (Rauluseviciute et al., 2020). Previous studies have shown that DNA methyltransferases were upregulated and histone modifications were modulated in prostate cancers (Patra et al., 2002; Seligson et al., 2005; Rauluseviciute et al., 2020). Although the molecular basis for the local rearrangement of histone marks (aberrant profiles of histone methylation marks) is diverse and poorly understood, studies suggest that aberrant expressions and *de novo* genetic mutations in histone modifiers, such as LSD1 (an H3K4 methytransferase) and EZH1/2 (the sole H3K27 methytransferasess), may play a role (Metzger et al., 2005; Duan et al., 2020). Overall, these findings indicate that there are several alterations in epigenetic events and the related regulatory functions during prostate cancer development. Since the transition of prostate cancer into CRPC is irreversible and is associated with AR-mediating androgen signaling, we focused on the functions of AR and its co-regulators in prostate cancer development in this review.

### 1.3 Androgen signaling in prostate cancer development and drug resistance

The prostate is a part of the male reproductive system and requires androgen for tissue development and maintenance (Kawano et al., 2003; Matsumoto et al., 2013). In addition to the male reproductive organs, the brain, skeleton, adipose, and several organs are targets of androgen, and treatment with androgen antagonists can cause side effects, such as hot flashes, bone loss, and weight gain (Grossmann and Zajac, 2011; Nguyen et al., 2015). Androgen serves as a prime endocrine male steroid hormone, and active androgens specifically bind to AR, increasing the accumulation of activated AR in the nucleus to direct gene regulatory program in a spatial- and temporal-manner. Unlike estrogen, which has two types of nuclear receptors, androgens have only one receptor (AR) in androgen signaling (Kawano et al., 2003; Matsumoto et al., 2013). The important role of AR in androgen signaling is supported by clinical observations in patients that are genetically deficient in AR function and in transgenic mouse models (Matsumoto et al., 2013; Hayakawa et al., 2022).

**FIGURE 1:**
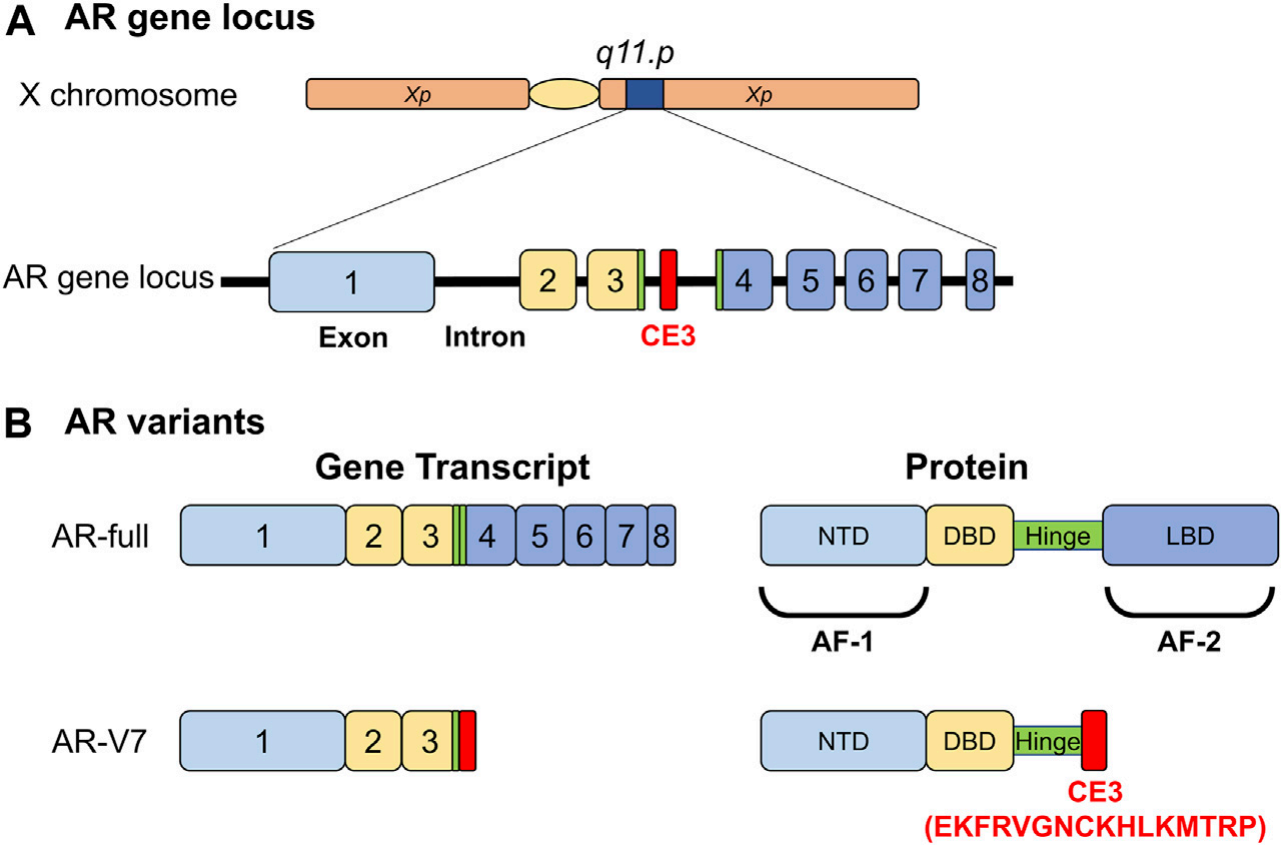
Functional structures of androgen receptor (AR) and its variant (AR-V7) selectively emerging in malignant prostatic tumors. **(A)** The human gene locus of the androgen receptor. CE; AR-V7- specific exon. **(B)** Functional structure of AR and AR-V7. NTD; N-terminal end: DBD; DNA-binding domain: LBD; Ligand-binding domain: AF-1; Autonomous transactivation function-1: AF-2; Autonomous transactivation function-2.

Accumulating evidence indicates that androgen signaling and AR expression play important roles in prostate cancer, even after the transition into the CRPC state. For example, an increase in AR gene expression and mutations in cancer driver genes, such as *PTEN, MYC,* and *TP53*, are often observed in progressive CRPC (Takeda et al., 2018; Viswanathan et al., 2018). In breast cancer, ERα is a clinical standard marker, and its loss predicts a transition into a drug-resistant state (Kuukasjärvi et al., 1996). In contrast, AR expression is detected in prostatic tumors even in the advanced stages, indicating that a loss of AR expression is not a clinical marker for prostate cancer. However, the AR mRNA variant AR-V7 is known to appear only during transition to CRPC (Henshall et al., 2001; Sharifi, 2013; Antonarakis et al., 2014). Since the AR-V7 protein does not possess a ligand binding domain and is assumed to act as a constitutively active receptor (Figure 1), it was recently characterized as an AR repressor (Cato et al., 2019), indicating that its expression inhibits AR-mediated androgen signaling in CRPC.

### 1.4 Androgen receptor function in androgen signaling

Androgen receptors are a pivotal factor in androgen signaling under physiological settings (Kawano et al., 2003; Matsumoto et al., 2013), as evidenced by phenotypic abnormalities in AR-deficient humans and mice (Kawano et al., 2003; Shiina et al., 2006; Matsumoto et al., 2013). Additionally, the importance of AR in the male external genitalia and reproductive organs, including the prostate, has been well illustrated in mouse lines with genetically disrupted AR gene (Kawano et al., 2003; Sato et al., 2004). Androgen receptors are important for developing and maintaining male reproductive organs in mammalians. Moreover, AR deficiency has been shown to affect other biological activities in humans and mice, including bone remodeling, energy consumption, and sexual behaviors (Hughes et al., 2012; Matsumoto et al., 2013). Accumulating evidence suggests that the androgen signaling pathway exerts its functions by mediating AR-related gene regulatory networks (genomic pathway); however, a non-genomic pathway *via* a cell membrane receptor has been hypothesized but is yet to be thoroughly studied. The active form of androgen circulating in mammalian serum is dihydrotestosterone (DHT), and the other forms serve as endogenous AR ligands with weak biological activities (Matsumoto et al., 2013; Bulun, 2014; Hayakawa et al., 2022). Most AR molecules are localized in the cytosol, but the binding of androgens translocates AR into the nucleus for gene regulation.

**FIGURE 2:**
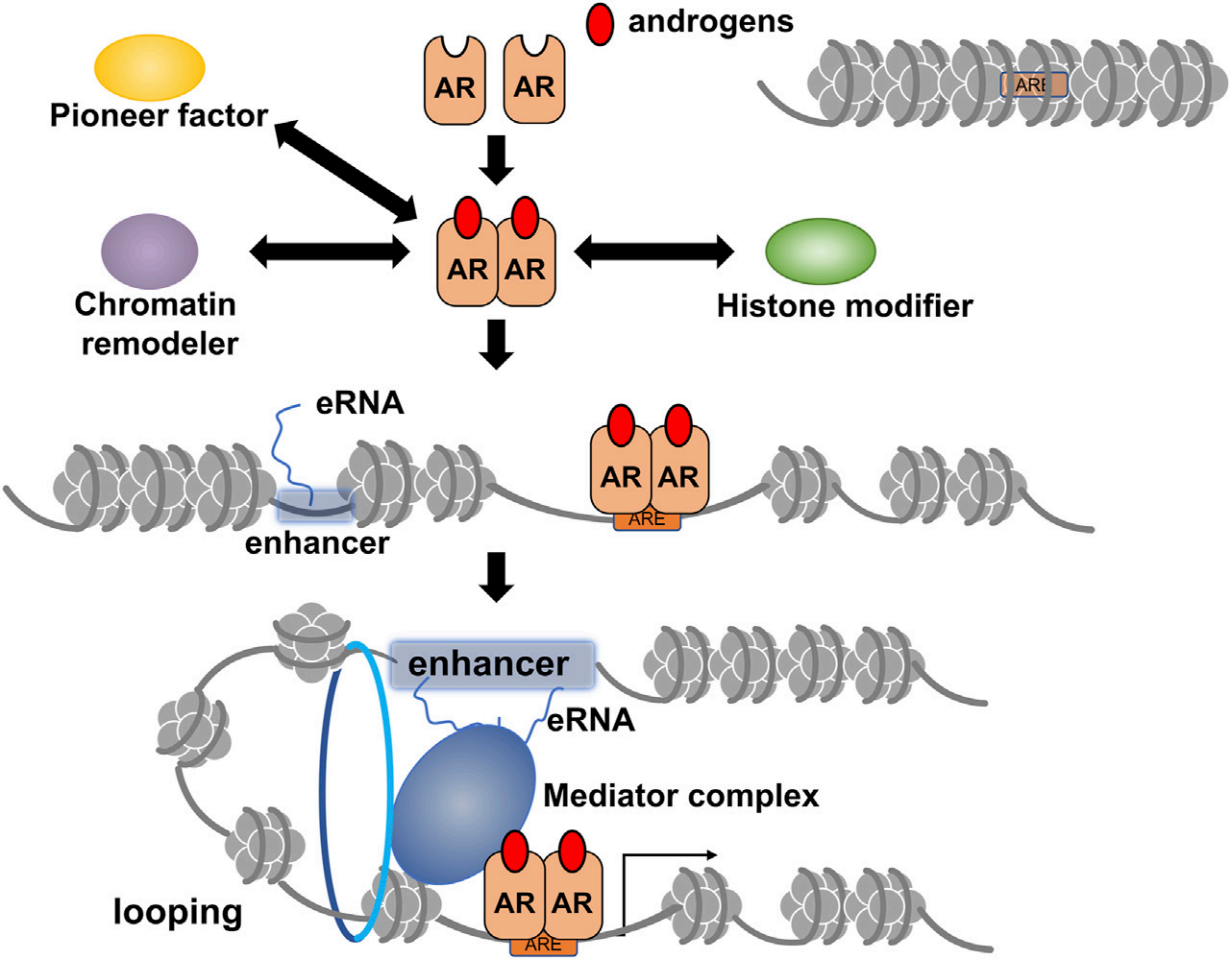
A schematic of ligand-dependent gene regulation by androgen receptor (AR) through epigenetic modifications and chromatin reorganization. The AR target site surrounding inactivated chromatin array is opened through chromatin remodeling by the activities of chromatin remodelers, histone modifiers, and pioneer factors, such as forkhead box A1 (FOXA1). Details of chromatin remodelers, histone modifiers, and transcriptional co-regulators involved in androgen-induced gene regulation by AR are described in Table 1. Formation of chromatin looping is induced for efficient transcription by activated AR, and enhancer RNA transcribed from potent enhancers, such as super-enhancer (SE), facilitates this process. Notably, it is currently unclear whether chromatin opening for AR DNA binding is associated with the eRNA-induced formation of chromatin looping.

Androgen receptors belong to the nuclear receptor (NR) superfamily consisting of 48 members in humans and act as DNA-binding transcription regulatory factors (Figure 1) (Mangelsdorf et al., 1995; Hayakawa et al., 2022). Similar to other NR members, the AR protein is divided into functional domains from A to E, with the central C domain composed of two zinc finger motifs necessary for recognizing and binding to the target DNA sequence. The N-terminal A/B and E domains encompass autonomous activation functions (originally named as AF-1 and AF-2 domains) and are regarded as the docking sites for AR co-regulators (Hayakawa et al., 2022). In the absence of AR agonists, direct and stable DNA binding of AR homodimer is evident in the androgen response enhancer element (ARE) composed of 5′-AGAACANNNTGTTCT-3′ motif or on the related sequences. However, *in vivo* whole genome analyses using chromatin immunoprecipitation sequence (ChIP-seq) and other approaches have shown that AR binds with chromatic DNA sequences that often encompass half of the core motif (5′-AGAACA-3′) of ARE (Lupien et al., 2008). AR binding sites are highly overlapped and adjacent to the binding site of forkhead box A1 (FOXA1). A pioneer factor such as FOXA1 is believed to remodel chromatin locally for efficient DNA binding of AR (Figure 2) (Lupien et al., 2008; Hayakawa et al., 2022). The chromatin environment surrounding AR binding sites is diverse (Chen and Dent, 2014); hence, FOXA1 acts as a pioneer factor for AR-dependent activation of gene expression and facilitates AR DNA binding (Lupien et al., 2008).

### 1.5 Androgen/AR target genes

Canonical AR target genes, including *KLK3* (coding PSA), *SGK*, and *TIPARP* (Bolton et al., 2007) are involved in exerting the biological actions of androgen. However, AR binding sites that have been successfully mapped by whole genome sequencing are mainly located in the intergenic regions, with more than 80% of the human genome harboring for non-coding RNAs (ncRNAs) (Cech and Steitz, 2014; Knoll et al., 2015). Some ncRNAs regulate gene expression at transcriptional and post-transcriptional levels. Among the several classes of ncRNAs, enhancer RNA (eRNAs) are transcribed by RNA polymerase II with high RNA turnovers from potent enhancers like super-enhancers (SEs) (Li et al., 2016b; Guan et al., 2018). Among enhancers facilitating AR-mediated transcription, a set of eRNAs was identified as androgen-inducible, and they promote androgen-induced expression of the AR target mRNAs by inducing chromatin looping for efficient transcription (Figure 2) (Hsieh et al., 2014; Nair et al., 2019; Sawada et al., 2021). Given the fact that the transcription of most ncRNAs is achieved using RNA polymerase II, the other classes of ncRNAs, such as long non-coding RNAs (lncRNAs), could be the targets of androgen-bound AR. Since 90% of the gene loci related to hereditary and chronic diseases are present in the ncRNA-coding regions of the human genome (Cech and Steitz, 2014; Knoll et al., 2015), these ncRNAs are believed to exhibit specific biological actions. Moreover, it is speculated that the biological activity of androgen is partly due to the functions of AR-regulated ncRNAs; however, further studies are necessary to validate this hypothesis.

### 1.6 AR co-regulators facilitate chromatin remodeling for gene regulation

Activated AR binds to chromatin through direct DNA binding or protein-protein interaction, depending on the chromatin environment (Kato et al., 2011; Hayakawa et al., 2022). If AR binding sites are within the activated chromatin-like euchromatic regions, rapid response to androgens in AR-mediated transcriptional regulation is achievable. Histone acetylation/deacetylation without dynamic chromatin reorganization is assumed to be sufficient for these transcriptional regulations (Hayakawa et al., 2022). In contrast, when the AR target sites are in the heterochromatinized regions, chromatin reorganization is required to facilitate AR binding using several AR co-regulators. FOXA1 binding motifs are often observed in the vicinity of AR binding sites. Since FOXA1 has an intrinsic function to remodel nucleosome arrays to facilitate AR binding (Figure 2), FOXA1 is regarded as a pioneer factor for AR-dependent activation of gene expression (Lupien et al., 2008). Two classes of AR co-regulators are involved in chromatin remodeling Table 1): histone modifiers and chromatin remodelers (Rosenfeld et al., 2006; Chen and Dent, 2014; Hayakawa et al., 2022). Both co-regulators are often present as large complexes of multiple subunits with protein motifs capable of recognizing modified histone residues. Direct and androgen-induced association of these co-regulators with AR has been eperimentally proven; moreover, the AR-coregulator complexes act as functional enzymatic units to modify histone marks (Rosenfeld et al., 2006; Kato et al., 2011; Chen and Dent, 2014). Reflecting the diversity of histone codes (histone modifications), several histone modifiers facilitating gene expression have been characterized (Rosenfeld et al., 2006; Kato et al., 2011; Chen and Dent, 2014), most of which are involved in the coregulation of AR function in androgen-regulated gene expression. Histone acetyltransferases (HATs) and histone deacetylases (HDACs) are considered effective histone modifiers for rapid gene regulation by AR (Rosenfeld et al., 2006; Kato et al., 2011). Apart from histone acetylation, a HAT (p300) was recruited to AR in an androgen-dependent manner, thereby acetylating AR to potentiate androgen-induced gene expression (Thompson et al., 2022). However, histone lysine methyltransferases (HKMTs) and histone demethylases (HDMs) act as pivotal AR co-regulators in instances where chromatin reorganization is requisite, consistent with the importance of methylated states of histone H3 K4, K9, K27 and K36 residues (Greer and Shi, 2012; Hayakawa et al., 2022). Additionally, several animal experiments have elucidated the roles of HKMTs and HDMs in prostate cancer development (Tong et al., 2016; Huang and Xu, 2017; Vatapalli et al., 2020). LSD1 was initially reported to act as an AR co-activator *via* demethylation of methylated H3K9 residue, along with upregulated expression of LSD1 in malignant prostate tumors (Metzger et al., 2005). However, the HDM activity of LSD1 for methylated H3K4 residue has been observed in other types of tumors (Hayami et al., 2011). Moreover, studies have shown a correlation between the expression levels of enzymes facilitating AR function in prostatic tumors and the malignancy of the tumor (Tong et al., 2016; Huang and Xu, 2017; Vatapalli et al., 2020). Accordingly, alterations in the expression levels and genetic mutations of the related enzymes were associated with life prognosis in prostate cancer patients (Tong et al., 2016; Huang and Xu, 2017; Vatapalli et al., 2020). Since the development of prostate cancer and the transition to the CRPC state are irreversible, the dynamic reorganization of the whole genome landscape is conceivable, and HKMTs and HDMs may regulate these epigenetic processes by redirecting histone code combinations.

**TABLE 1:** List of AR co-regulators.

	Co-regulator	Function	Type/mechanism of a co-regulator	References
Histone modifier	NSD2 (MMSET, WHSC1)	H3-K4,9,27,36 methylation	AR translocation into nucleus	(Kang et al., 2009; Ezponda et al., 2013)
	EZH2 (KMT6A)	H3-K27 trimethylation	Indirect protein partner of AR	Asangani et al., 2013; Yang and Yu 2013; Park et al., 2021
	SUV39H2 (KMT1B)	Adjacent to AR WXXLF motif	Direct protein partner of AR	(Askew et al., 2017)
	SETDB1 (KMT1E)	H3-K9 methylation	Silencing AR gene	Cho et al., 2014; Lazaro-Camp et al., 2021
	SMYD3 (KMT3E)	H3-K4 methylation	Upregulation of AR expression	(Liu et al., 2013)
	PRMT1 (HMT2)	Facilitating AR biding at enhancer elements	Enhancing AR signaling	Wang et al., 2001; Tang et al., 2022
	PRMT4 (CARM1)	H3-R17 methylation	Enhancing AR signaling	Schurter et al., 2001; Kang et al., 2004
	PRMT5	H4-R3 dimethylation	Enhancing AR signaling	(Deng et al., 2017)
	SET9 (KMT5)	Methylation of AR at K632	Enhancing AR signaling	(Gaughan et al., 2011)
	DOT1L	H3-K79 methylation	Direct protein partner of AR	(Vatapalli et al., 2020)
	G9a (KMT1C, BAT8, GAT8)	H3-K9 methylation	Upregulation of AR expression	(Lee et al., 2006)
	MLL	H3-K4 methylation	Direct protein partner of AR	(Malik et al., 2015)
	HAT1(KAT1)	H4-K5 and H4-K12 acetylation	Upregulation of AR expression	(Hong et al., 2021)
	TIP60 (KAT5)	Acetylation of AR at K630, 632 and 633	Enhancing AR signaling	Gaughan et al., 2002; Tan et al., 2020
	p300/CBP	Acetylation of AR at K630, 632 and 633	Enhancing AR signaling	Fu et al., 2000; Lasko et al., 2017; Jaiswal et al., 2022
	PCAF (KAT2B) (P300/CBP-Associated Factor)	Acetylation of AR at K632 and 633	Enhancing AR signaling	(Fu et al., 2000)
	KAT2A (GCN5)	Acetylation of AR at K630	AR translocation into nucleus	(Lu et al., 2021)
	ARD1	Acetylation of AR at K618	Enhancing AR signaling	(Kuhns et al., 2018)
	P160 SRCs	Forming common coactivators complex	Enhancing AR signaling	(Xu et al., 2009a)
	RNF6	Ubiquitination of AR at K845 and 847	Enhancing AR signaling	(Xu et al., 2009b)
	RNF20, RNF40	Ubiquitination of H2B-K120	Enhancing AR promoter activity	(Jääskeläinen et al., 2012)
	USP10	Deubiquitylation of H2A.Z	Enhancing AR signaling	Faus et al., 2005; Draker et al., 2011
	PKN1 (WDR5)	Phosphorylation of H3-T11	Enhancing AR signaling	(Kim et al., 2014)
	LSD1 (KDM1A)	H3-K4 and K9 demethylation	Enhancing AR signaling	Metzger et al., 2005; Kahl et al., 2006; Hayami et al., 2011
	JMJD1A (KDM3A)	H3-K9 demethylation	Enhancing AR signaling	(Yamane et al., 2006)
	JMJD2B (KDM4B)	Binding PLK1 promoter	Enhancing AR signaling	Coffey et al., 2013; Duan et al., 2015
	JMJD2C (KDM4C)	H3-K9 demethylation Colocalizing with LSD1 and AR	Enhancing AR signaling	(Wissmann et al., 2007)
	JARID1B (KDM5B)	H3-K4 demethylation	Enhancing AR signaling	(Xiang et al., 2007)
	JARID1C (KDM5C)	H3-K4 demethylation	Associating with a reduced PSA relapse-free survival	(Stein et al., 2014)
	JARID1D (KDM5D)	H3-K4 demethylation	Suppressing AR signaling	Li et al., 2016a; Komura et al., 2016
	KDM6B	H3-K27 demethylation	ARs suppress KDM6B transcription	Cao et al., 2021; Yıldırım-Buharalıoğlu 2022
	PHF8 (KDM7B)	H3-K9 and H4-K20 demethylation	Enhancing AR signaling	(Tong et al., 2016)
	JMJD5 (KDM8)	Activation of ARE-driven promoters	Enhancing AR signaling	(Wang et al., 2019)
	HDAC1 (classI)	Facilitating corepressor	Inhibiting of AR signaling	Gaughan et al., 2002; Fu et al., 2003
	HDAC7 (classIIa)	Deacetylation of AR at K630	Inhibiting of AR signaling	(Zhang et al., 2022)
	HDAC6 (classIIb)	Deacetylating HSP90	Enhancing AR signaling	Bali et al., 2005; Chen et al., 2005; Gibbs et al., 2009
	SIRT1 (classIII)	Deacetylation of H3 in AR-dependent gene promoters	Inhibiting of AR signaling	Fu et al., 2006; Dai et al., 2007
Chromatin remodeler	CHD1	Regulating HOXB13 enriched AR cistrome		(Augello et al., 2019)
	CHD8	AR localization to the TMPRSS2 enhancer		(Menon et al., 2010)
	SMARCA2 (BRM)	Modulating DNA accessibility		(Sun et al., 2007)
	SMARCA4 (BRG1)			Sun et al., 2007; Ding et al., 2019; Giles et al., 2021
eRNA	KLK3e	Chromosomal looping		(Hsieh et al., 2014)
Other	Cyclin E	Enhancement of AF-1 transactivation function		(Yamamoto et al., 2000)

Chromatin remodeling complexes conduct actual chromatin remodeling in an ATP-dependent manner (Chen and Dent, 2014). Hence, each complex contains ATPase as a critical driver for chromatin remodeling for gene regulation, although several complexes have distinct ATPases. Multi-faceted surfaces of the remodeler complexes may be advantageous in protein-protein interaction owing to multiple subunit assemblies (Kato et al., 2011; Hayakawa et al., 2022), enabling the complexes to stably and transiently associate with chromatin and other AR co-regulators (Figure 2). Among the remodeler complexes, direct interaction of AR with two types of SWI/SNF complexes has been reported (Ding et al., 2019; Cyrta et al., 2020). Recently, the pivotal role of this complex in AR-mediated prostatic tumor development was demonstrated using a compound (AU-15330) capable of selective degradation of SWI/SNF ATPases (Xiao et al., 2022). Treatment with AU-15330 induced selective proteolysis of the core subunits (SMARCA2 and SMARCA4) and promoted the dislodging of AR and FOXA1 from chromatin in prostate cancer cell lines and suppressed the growth of xenograft prostatic tumors in mice. Additionally, combined treatment with AU-15330 and a clinically used AR antagonist (enzalutamide) successfully reduced tumor volume within 3 months (Xiao et al., 2022). Overall, these results suggest that dynamic chromatin reorganization is involved in prostate tumor progression.

### 1.7 Enhancer RNA (eRNA) as AR co-regulator for gene regulation

Locally looped chromatin between the promoter region and enhancer(s) harboring AR binding sites in the target gene loci are important in initiating efficient transcription in response to androgens. Consistently, AR bindings have been identified in the multiple sites over the enhancers of the *KLK3* and *KLK4* (Hsieh et al., 2014; Sawada et al., 2021; Takayama et al., 2021). Recent findings suggest that ncRNAs transcribed from potent enhancers, such as SEs, assist in looping chromatin with the aid of mediator and co-cohesion complexes (Li et al., 2016b; Guan et al., 2018; Nair et al., 2019). Notably, a class of ncRNAs known as androgen-inducible eRNAs facilitates androgen-induced prostate-specific antigen [PSA (*KLK3*)] gene expression in human prostate cancer cell lines (Hsieh et al., 2014; Sawada et al., 2021). Moreover, androgen-inducible eRNAs have been reported to act as a trigger to form a massive transcription initiation complex with chromatin looping *via* a liquid-liquid phase separation (LLPS) state (Figure 2) (Takayama et al., 2021). The transcription initiation complex contains AR, eRNAs, mediator complex components, and HAT CBP/p300, indicating that the complex also serves as a histone modifier unit. Additionally, this vast complex may combine with a canonical transcription initiation complex consisting of a mediator complex, fundamental transcription factors, and RNA polymerase II. Overall, eRNA may be considered an RNA co-regulator for AR in addition to acting as a protein transcription co-regulator (Hsieh et al., 2014; Sawada et al., 2021; Takayama et al., 2021).

### 1.8 Mode of action of the clinically applied AR antagonists

Since prostate cancer is androgen-dependent at early stages, androgen-deprivation therapy is currently used in patients. The most common therapy is based on enzyme inhibitor treatments and AR antagonists (Sumanasuriya and De Bono, 2018), one of which is an inhibitor of a P450 enzyme (*CYP17*) converting precursors into steroids, leading to the deprivation of locally produced androgen in prostatic tumors (Matsumoto et al., 2013). These enzyme inhibitors are effective as chemical androgen inhibitors, with fewer side effects than other enzyme inhibitors and orchidectomy.

AR antagonists can be classified into two classes, with both classes exhibiting antagonistic actions against endogenous androgens in terms of activation of AR function. However, a recently developed AR antagonist called enzalutamide acts by blocking AR translocation from the cytosol to the nucleus, irrespective of AR binding, in a manner different from the mode of action of the canonical AR antagonists flutamide and bicalutamide (Scher et al., 2012; Sumanasuriya and De Bono, 2018). Thus, enzalutamide is regarded as a functional inhibitor of AR by blocking nuclear transport. The canonical AR antagonists are competitive in androgen binding and inhibitory for the transactivation function of AR. However, these antagonists are incapable of blocking nuclear transport coupled with ligand binding-induced alteration(s) in AR structure. The clinical outcomes and gene expression profiles of prostate cancer patients support the clinical benefits of these two types of AR antagonists (Sumanasuriya and De Bono, 2018; Rice et al., 2019). However, studies are yet to elucidate why prostatic tumors successfully treated with chemical therapy are prone to be malignant after specific periods (Pimenta et al., 2022).

### 1.9 Chromatin remodeling by AR synthetic ligands

**FIGURE 3:**
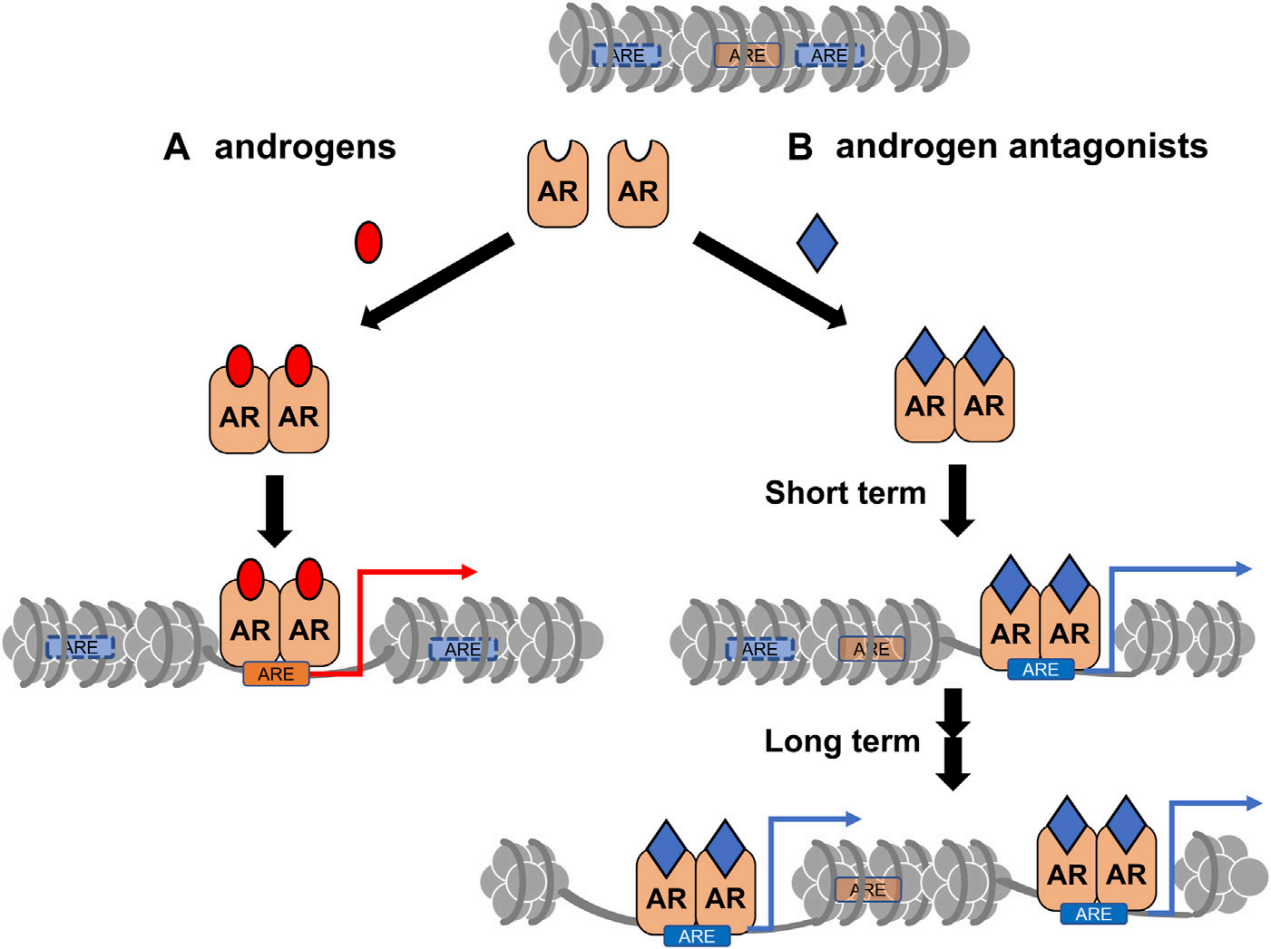
A schematic of differential chromatin remodeling by androgens vs. androgen receptor (AR) antagonists. **(A)** The canonical remodeling by AR-bound androgens (red). **(B)** The canonical target sites (ARE with orange) for AR-bound androgens could be closed by chromatin reorganization induced by the antagonist (blue) -bound AR. Cryptic AR target sites (ARE with blue) could be opened for AR binding.

**FIGURE 4:**
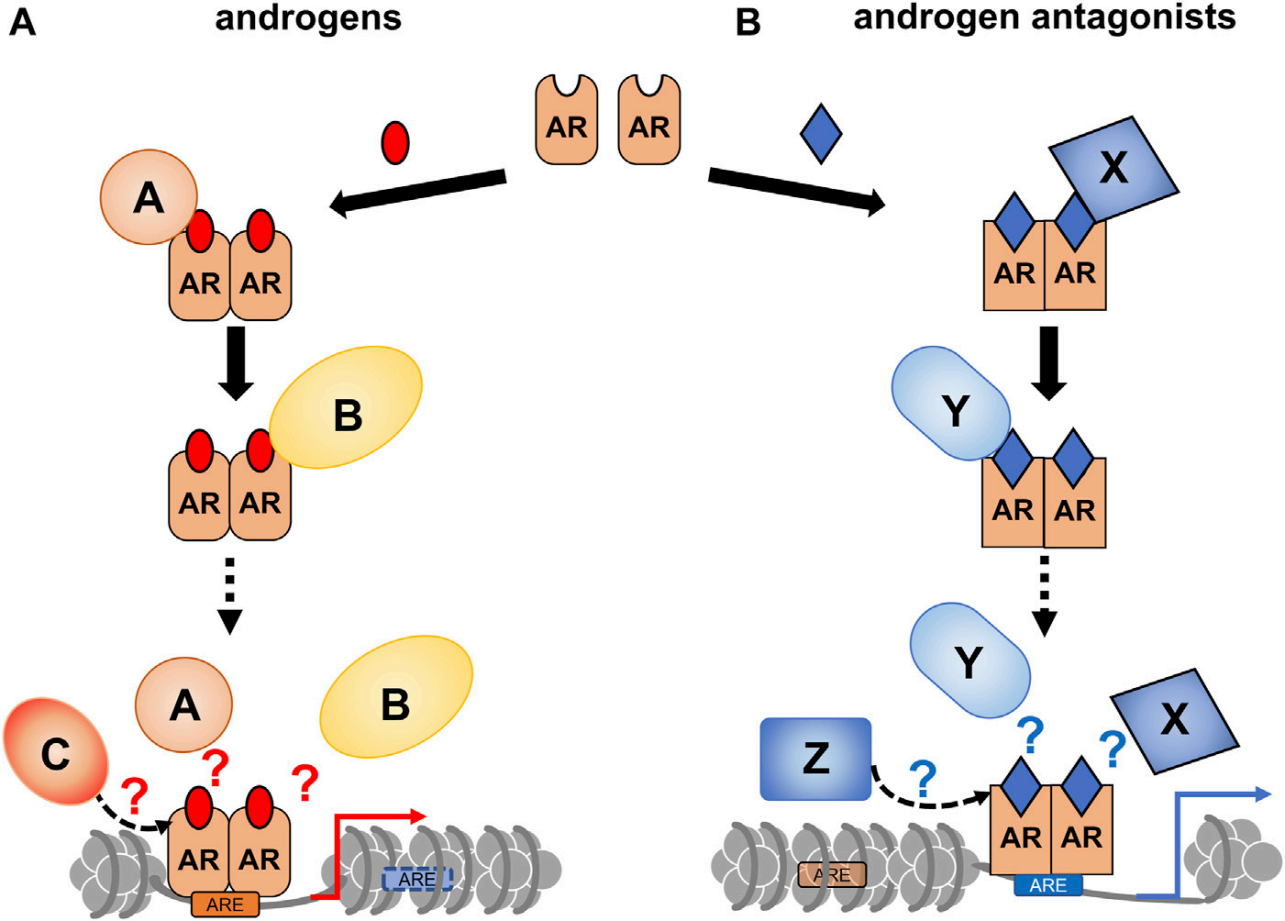
A schematic of androgen receptor (AR)-coregulator-mediated chromatin remodeling by ligand binding. A ligand type-specific set of AR co-regulators facilitate ligand type-specific chromatin reorganization. Associations of AR with co-regulators are differentially induced by AR ligands. In order to reorganize the chromatin environment, multiple co-regulators appear to be transiently and sequentially recruited for ligand-bound AR. **(A)** The canonical recruitment of co-regulators by androgen binding is depicted. **(B)** Androgen antagonists are potent to recruit non-canonical co-regulators, thereby leading atypical chromatin remodeling.

CRPC often develops after androgen deprivation therapy, and the acquired hormone resistance is irreversible (Scher et al., 2012; Attard et al., 2016; Li et al., 2016c). Based on changes in gene expression profiles during the progression of prostate tumors (Wang et al., 2009; Cyrta et al., 2020), it is speculated that the transition to the CRPC state is highly associated with chromatin reorganization. Similar to other types of cancer, epigenetic regulators are involved in prostate cancer, and their malfunction has been well-documented (Gao and Alumkal, 2010; Thompson et al., 2022). Recently, the effect of a canonical AR antagonist (bicalutamide: Bic) in chromatin remodeling and expression profile in a human prostate cancer cell line (LNCaP cells) was examined (Sawada et al., 2022). ATAC-seq analysis showed that Bic-induced rearrangement pattern of the chromatin array was different from DHT-induced rearrangement (Figure 3), indicating that Bic is also effective in remodeling the chromatin array. Additionally, Bic acted as a transcriptional antagonist for AR function (Sawada et al., 2022). Although it is unclear if other AR antagonists are capable of remodeling the chromatin array, clinical studies have shown that these treatments modulate the chromatin landscape. In this respect, further studies of the epigenetic actions of the AR antagonists are necessary to improve the understanding of the molecular mechanism of the transition from a hormone-dependent to hormone-resistant state in prostatic tumors (Figure 4).
